# Compliance with minimum information guidelines in public metabolomics repositories

**DOI:** 10.1038/sdata.2017.137

**Published:** 2017-09-26

**Authors:** Rachel A. Spicer, Reza Salek, Christoph Steinbeck

**Affiliations:** 1 European Bioinformatics Institute (EMBL-EBI), Hinxton, Cambridge CB10 1SD, UK; 2 Friedrich-Schiller-University, Fürstengraben 1, 07743 Jena, Germany

**Keywords:** Metabolomics, Standards, Research data

## Abstract

The Metabolomics Standards Initiative (MSI) guidelines were first published in 2007. These guidelines provided reporting standards for all stages of metabolomics analysis: experimental design, biological context, chemical analysis and data processing. Since 2012, a series of public metabolomics databases and repositories, which accept the deposition of metabolomic datasets, have arisen. In this study, the compliance of 399 public data sets, from four major metabolomics data repositories, to the biological context MSI reporting standards was evaluated. None of the reporting standards were complied with in every publicly available study, although adherence rates varied greatly, from 0 to 97%. The plant minimum reporting standards were the most complied with and the microbial and *in vitro* were the least. Our results indicate the need for reassessment and revision of the existing MSI reporting standards.

## Introduction

Leading members of the metabolomics community organised the development of the Metabolomics Standards Initiative (MSI), following examples from other communities^1,2^, in 2005. This work built on earlier efforts by the Standard Metabolic Reporting Structure initiative^3^ and the Architecture for Metabolomics consortium (ArMet)^4^. Under the umbrella of the MSI^5,6^ working groups for biological context metadata, chemical analysis, data processing, ontology and data exchange were formed. These working groups published a series of reports, with minimal reporting standard recommendations for each area. The MSI reports were summarized by Goodacre^7^. For the following 6 years, the recommendations guided the data collection in individual labs and specialised databases^8,9^. Since 2012, the first general purpose, global repositories for metabolomics data, the European Bioinformatics Institute’s MetaboLights^10^ and United States Government National Institute of Health (NIH) Metabolomics Workbench^11^ have been launched. During the early phase of this emerging network of global data exchange, the COordination of Standards in MetabOlomicS (COSMOS) consortium^12^ was instrumental in filling some of the gaps in data standards and data formats. Additionally, the MetabolomeXchange consortium that was based upon the successful ProteomeXchange^13^ was founded through the COSMOS consortium.

Four metabolomics data repositories have been developed to fulfil the MSI guidelines for minimum metadata reporting: MetaboLights^10^, Metabolomics Workbench^11^, Metabolomics Repository Bordeaux (MeRy-B)^9^ and Metabolic Phenotype Database (MetaPhen)^14,15^ (Table 1). The global repositories MetaboLights^10^ and Metabolomics Workbench^11^ accept data across a wide variety of instrumental data formats and species. Metabolomics Workbench additionally includes the ability to perform exploratory statistical analysis on publicly available data sets. Compared to Metabolomics Workbench, MetaboLights has a greater focus on curation and has stricter submission guidelines. MeRy-B is a small repository, exclusively for plant metabolomics datasets. It focuses on proton nuclear magnetic resonance (^1^H-NMR) data, but also hosts gas chromatography—mass spectrometry (GC-MS) data. These three repositories are all data providers for the MetabolomeXchange consortium. MetaPhen is part of MetabolomeExpress, a GC-MS metabolomics data analysis platform. The majority of the studies in MetaPhen are of plants and are GC-MS based. MetabolomeExpress is a web-server, providing processing, statistical analysis, visualisation and data storage. A newer metabolomics repository is Global Natural Products Social Molecular Networking (GNPS)^16^, which focuses on natural products. As it has no requirements for reporting experimental metadata and does not aim to fulfil MSI guidelines, data from this repository has not been included in this research.

Due to the emergence of this global network for metabolomics data exchange and dissemination, the scientific community now has access to hundreds of public datasets for re-analysis and reuse. Data sharing benefits the community by improving reproducibility and signalling credibility of the data; researchers who make their data publicly available demonstrate confidence in their research^17^. This is now widely recognised by funders, learned societies and scientists themselves and the fundamentals of good data sharing practises have recently been revisited under the catchy acronym FAIR, which stands for data being Findable, Accessible, Interoperable and Reproducible^18^. The importance of FAIR data sharing practices are highlighted by a recent nature survey which found that two thirds of researchers are concerned about reproducibility^19^. Researchers also directly benefit from sharing their data—studies that have data publicly available in a repository receive more citations than those without publicly available data^20^. Examples of reuse of publicly available data from the MetaboLights repository include research into retention time prediction^21^ and evaluation of the impact of normalisation methods^22^ and scaling^23^ on metabolomics data. Increasingly, journals and public funding bodies are requiring researchers to publish their data, which will lead to further growth in the number of publicly available studies in the coming years.

Easy to use reporting standards aid researchers in publishing their data^24^. Good reporting standards also ensure consistency of metadata between datasets, and facilitate data reuse and data merger across studies. Conversely, poor reporting standards are a burden to data reuse and do not reflect current community needs^25^. Here, we have inspected the currently available public data sets in four metabolomics repositories for their compliance with existing biological context MSI reporting standards in order to assess how timely the MSI standards still are.

## Results

In order to evaluate the compliance to the MSI standards three species commonly studied in biological sciences, which were prevalent across the repositories, and encompassed four of the five MSI guidelines, were selected for analysis. Of the four analyzed repositories, only the general purpose MetaboLights^10^ and Metabolomics Workbench^11^ contained studies representing all three of the studied species: *Homo sapiens, Mus musculus* and *Arabidopsis thaliana.* Neither of the plant focused repositories MetaPhen^14,15^ or MeRy-B^9^ contained *M. musculus* studies, although MetaPhen contained a single *H. sapiens* study. In total 399 studies were analysed.

### MSI Guidelines

The MSI biological context metadata working group, consisting of four subgroups, produced metadata guidelines for the following biological experiment areas: *Mammalian/ in vivo*^26^*, Microbial and in vitro*^27^, *Plant*^28^ and *Environmental*^29^. The Mammalian/ *in vivo* report is split into two sets of reporting standards: *Mammalian Clinical Trials and Human Studies* and *Pre-clinical*. Each report consists of the minimum reporting standards required for each class of experiment, as agreed upon by the MSI subgroup. These standards consist of all the descriptive information about an experiment (the metadata) that was considered crucial to the understanding of the data, in order to enable replication of the experiment and re- or further data analysis^5^. As well as the minimal reporting standards, the *Mammalian Clinical Trials and Human Studies* (*Clinical* for brevity) and *Pre-clinical* reports also included recommended further information. Additional best practice reporting standards were also included in the *Microbial and in vitro* report (abbreviated to *in vitro*). The number of metadata mandated by each minimal and optional or best practice reporting standard was quantified (Table 2), with some guidelines being combined to obtain a binary list (e.g. the organ and cell type standards in the *Plant* guidelines were combined to a single item, as studies usually include only one of these biosources). None of the repositories contained a sufficient number of environmental studies to enable testing of compliance to the environmental set of reporting standards.

### Meta-analysis

Meta-analysis of the data shows that there are no reporting standards that are complied with in every publicly available study (Supplementary Tables 1–7). The overall rate of compliance across repositories varies from 0–97%. However, there are reporting standards that are complied with by every study within a repository (Supplementary Tables 3–4, 6). Across all of the guidelines, reporting standards relating to biosource had the highest percentage compliance.

**Figure 1:**
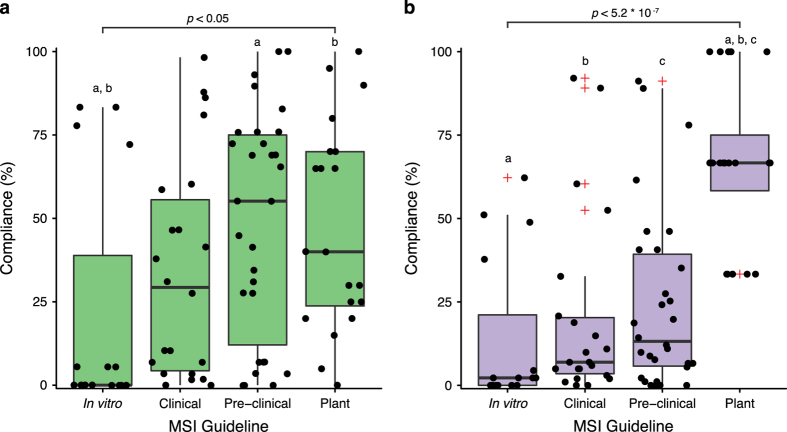
Compliance to the MSI minimum reporting standards. Combined box-and-whisker and dot plots showing the percentage compliance with the MSI minimum reporting standards within the (**a**) MetaboLights and (**b**) Metabolomics Workbench repositories. Red ‘+’ indicate outliers; each ‘dot’ represents compliance to a single reporting standard. Letters denote significant differences in compliance (Kruskal Wallis, Dunn *post-hoc* test with Benjamini-Hochberg correction).

In MetaboLights (ML) the *in vitro* minimal reporting standards had significantly lower (Kruskal-Wallis χ^2^=9.37, *df*=3, *P*<0.05) compliance compared to the pre-clinical and plant minimal reporting standards (Fig. 1a). The plant minimum reporting standards were adhered to at a significantly higher rate than the three other guidelines: *in vitro*, clinical and pre-clinical (Kruskal-Wallis χ^2^=32.05, *df*=3, *P*<5.2×10^−7^) in Metabolomics Workbench (MW) (Fig. 1b). Of all the studied minimal reporting standards, *in vitro* had the lowest compliance in both repositories. Conversely, the best practice *in vitro* reporting standards had significantly higher compliance than the optional clinical and pre-clinical guidelines in MW (Supplementary Fig. 1b) and significantly greater compliance than clinical in ML (Supplementary Fig. 1a).

It was also found that some of the clinical studies in the repositories did not fully report the metadata collected in the study. Instead the reporting standards were partially complied with by the reporting of ‘implicit’ metadata. Rather than reporting, for example gender, as a factor for each individual sample, the metadata were reported as descriptive statistics in the corresponding publication to the study. In 32.76% of clinical studies in ML and 6.93% in MW, gender was reported as implicit metadata. Both ethnicity (ML: 13.79%, MW: 1.98%) and disease status (ML: 5.17%, MW: 3.96%) were also found to be reported as implicit metadata in a number of studies.

**Figure 2:**
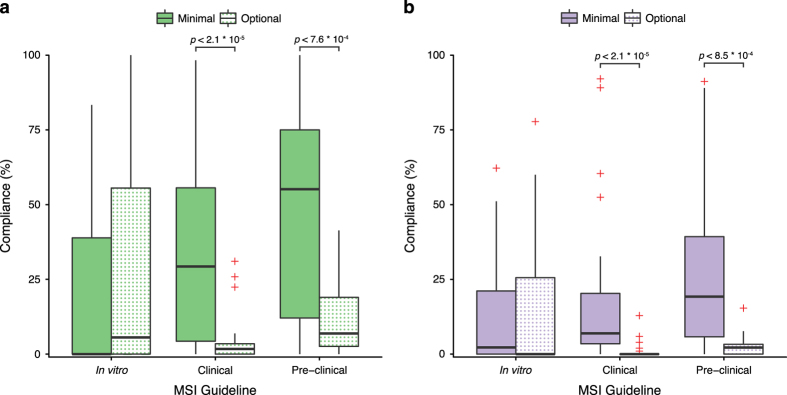
Comparison of Compliance to Minimal and Best Practice Reporting Standards. Box-and-whisker plots showing the percentage compliance to the minimal and optional/best practice reporting standards in (**a**) MetaboLights and (**b**) Metabolomics Workbench. Minimal reporting standards are indicated with filled bars, optional/best practice reporting standards are indicated with a dot pattern and red ‘+’ indicate outliers. Mann-Whitney U tests were used to assess significance.

There was significantly greater compliance to the minimal reporting standards compared to the optional for the clinical and pre-clinical guidelines within the ML (Mann-Whitney *U* test, clinical: *U=*118.5, *P*<2.1×10^−5^, pre-clinical: *U*=94, *P*<7.6×10^−4^) and MW repositories (Mann-Whitney *U* test, clinical: *U=*78.5, *P*<2.1×10^−5^, pre-clinical: *U*=384.5, *P*<8.5×10^−4^). However, no significant difference was found between the compliance with the minimal and best practice *in vitro* reporting standards (Fig. 2).

**Figure 3:**
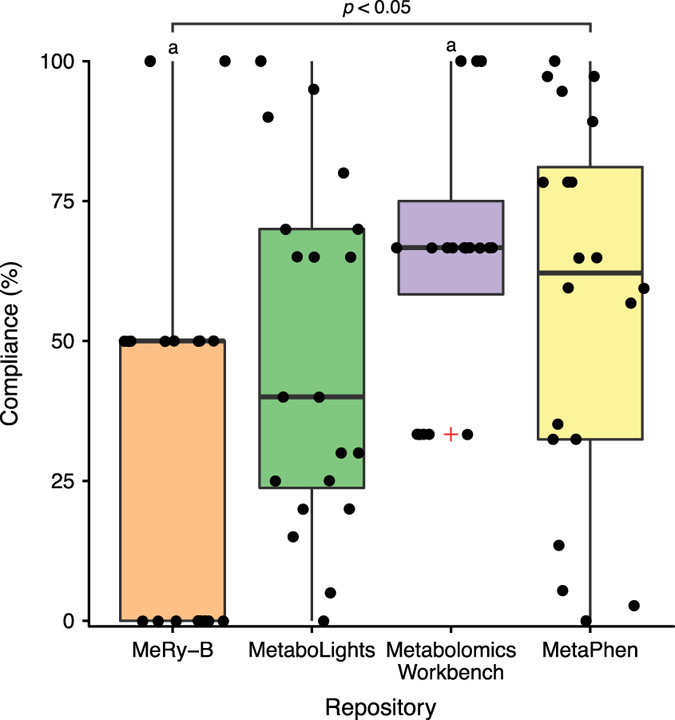
Compliance to Plant MSI Reporting Standards. Combined box-and-whisker and dot plots showing the percentage compliance of *A. thaliana* studies to the plant MSI minimum reporting standards within the MeRy-B, MetaboLights, Metabolomics Workbench and MetaPhen repositories. Red ‘+’ indicate outliers; each ‘dot’ represents compliance to a single reporting standard. Letters denote significant differences in compliance (Kruskal Wallis, Dunn *post-hoc* test with Benjamini-Hochberg correction).

Across the four repositories there were significantly different levels of compliance to the plant guidelines (Kruskal-Wallis χ^2^=8.38, *df*=3, *P*<0.05) (Fig. 3). Overall, differences in compliance across the four sets of reporting standards between ML and MW were not significant (Kruskal-Wallis χ^2^=2.55, *df*=3, *P*=0.11).

## Discussion

Our results show that the level of compliance to the different sets of reporting standards varies greatly across public repositories. Overall the plant minimal reporting standards had the highest rate of compliance and the microbial and *in vitro* had the lowest. The greater compliance to the plant guidelines may in part be due the preciseness of their wording. Whilst all of the MSI biological context subgroup reports encourage the use of ontologies, the plant report details precisely which ontology should be used for the entire description of the biosource, including species, genotype, organ and cell type^28^. This specificity aids ease-of-use. As there are only two *A. thaliana* studies in MeRy-B and three in MW, this biases the Kruskal-Wallis test and the significant difference between compliance to the plant reporting standards may result from this low sample size.

Whilst similar levels of compliance to minimal and best practice microbial and *in vitro* reporting standards may be due to the very low median compliance to the minimal standards, this may also stem from how minimal and best practice reporting standards are defined by the sub-working group. Only metabolomics specific factors are included in the minimal reporting standards, whilst all other general aspects, as well as additional factors specific to metabolomics experiments, are included in the best practice reporting standards. This means that *Cell Type* and *Treatment* are included as best practice and not minimal reporting standards. In all other sub-groups’ guidelines the equivalent reporting standards are included as minimal rather than best practice. This discrepancy between guidelines should be revisited in subsequent revisions to the MSI standards. It also supports the use of continuous revisions, as has been adopted by the proteomics community for MIAPE^2^.

Lower levels of compliance to the microbial and *in vitro* minimal reporting standards compared to the other sets of standards may result from researchers not understanding precisely what they are expected to report. There is much ambiguity, for example the stability reporting standard asks ‘What is known about the stability of (specific) metabolites during quenching, extraction and sample preprocessing?’^27^.

Differences in the rates of partial adherence with guidelines by reporting ‘implicit metadata’ between MetaboLights and Metabolomics Workbench may be accounted for by the percentage of studies included in the analysis that had an associated publication. Only 12% of human clinical studies in Metabolomics Workbench had an associated publication, in contrast to 96% of those in MetaboLights that did. This is also true for mouse pre-clinical studies, where 30% of Metabolomics Workbench studies had an associated publication, whereas 93% of MetaboLights studies did.

In specific instances e.g. gender in clinical *H. sapiens* studies (Supplementary Table 1) and age at study start in pre-clinical *M. musculus* studies (Supplementary Table 3) differences in compliance between MetaboLights and Metabolomics Workbench may result from MetaboLights more stringent submission guidelines.

Ultimately enforcing compliance to reporting standards is the responsibility of journals and data hosts^30^. If repositories do not comply with a standard, it will not be successful^31^. However, adhering to an entire set of reporting standards is time-consuming and submitters may be put off sharing their data by ambiguous wording or difficulty in obtaining metadata. Repositories therefore have a trade-off between attracting users to submit their data and enforcing reporting standards to ensure deposited data is informative. This could partially be addressed by systematically capturing experimental meta-data via Laboratory Information Management Systems (LIMS) during experiments as proposed by Rocca-Serra *et al.*^24^, or by tools such as mzML2ISA^32^ that can generate partially filled reporting templates for MetaboLights.

The need for data sharing and reuse is now very well established^18^ and has been adopted and promoted by all major funders^33–36^ and learned societies. In particular untargeted metabolomics holds the promise of correlating complex patterns—molecular phenotypes—of concentration changes and occurrences of metabolites with aspects of the exposome of an organism. In order to fulfil this promise and to enable discovery of these patterns, we will inevitably need more metadata on the exposome than most individual researchers will initially envision when designing an experiment. Furthermore, a number of initiatives to establish computational e-infrastructures for metabolomics have recently been funded, such as PhenoMeNal (http://phenomenal-h2020.eu) and MetaboFlow (http://europepmc.org/grantfinder/results?kw=MetaboFlow&page=1). These computational infrastructures aim to provide well-tested and reproducible workflows for metabolomics, where data, often from public repositories, is handed from one workflow node to the next for different steps of data processing. Such computational workflows critically depend on the public availability of data, well defined and open data formats and compliance with a given set of minimum information standards.

Considering that FAIR data sharing is not an end in itself but a means to enable better science and new scientific discoveries, our results indicate a need for a) a second round of MSI consultations where the existing standards are critically revisited and revised and b) that maintainers and curators of existing global repositories work with publishers, funders and most importantly their users and submitters to ensure completeness of data and metadata adhering to the existing standards. All metadata that are required for the reanalysis and reuse of data must be captured in the updated reporting standards, to ensure the maximum amount of information can be extracted from the data. We hope that our results will help us convince the global metabolomics community of these needs, so that we can be instrumental in facilitating the necessary next steps leading to revised MSI standards.

## Methods

In this research we have examined publicly available datasets to investigate their compliance with the MSI standards. Three species with the greatest number of publicly available studies across the four repositories were selected: *Homo sapiens, Mus musculus* and *Arabidopsis thaliana* (Supplementary Fig. 2). Four out of the five biological experimental areas of the MSI standards are covered by studies of these species. There are also *H. sapiens* and *M. musculus* studies that do not fit within the scope of any of the existing MSI standards, such as those of intra-laboratory differences or the development of new experimental techniques.

Every study from the four repositories including the selected species was first categorized by applicable biological context standards. Some studies contain multiple assays, which can include multiple species or both cell lines and clinical research. Multiple sets of MSI reporting standards can therefore be applicable to a single study. For files detailing how *H. sapiens* and *M. musculus* studies were classified please see Data Citation 1.

Following classification, every study was examined for its compliance with the reporting of each metadata included in the MSI guideline. For metadata to be considered reported, the metadata must be either directly included in the repository with the data or in a publication assessable by direct link from the study page. Raw data rating each metadata as either reported in accordance with the MSI guideline, or not reported in accordance to the guideline are available in Data Citation 1.

Some metadata were also recorded as ‘implicit’, where the metadata were not reported on a per sample basis, instead being reported as descriptive statistics for the entire study. Once adherence to each metadata reporting standard had been assessed for each individual study, the percentage of studies the metadata was reported in was calculated. The distribution of reporting of *minimal/ best practice* was found for each set of standards for each repository.

### *Homo sapiens*

Two of the MSI biological metadata reporting standards are applicable to *H. sapiens: Mammalian Clinical Trials and Human Studies and Microbial and in vitro*. Human clinical trials are classified as *Mammalian Clinical Trials and Human Studies* and *H. sapiens* cell line studies are categorized as Microbial and *in vitro* studies. There are also *H. sapiens* studies that are neither of these experiment types and are classified as *Other*.

There are 83 public *H. sapiens* studies in MetaboLights as of 17/03/07, 147 with the species ‘human’ in Metabolomics Workbench and 1 *H. sapiens* study in MetaPhen. As there was only a single *H. sapiens* study in MetaPhen, it was excluded from this research. Following classification there were 58 *Clinical*, 18 *in vitro* and 7 *Other H. sapiens* studies in MetaboLights. Human Metabolomics Workbench studies consisted of 99 *Clinical*, 45 *in vitro* and 3 *Other*.

### *Mus musculus*

The appropriate reporting standards for use with *M. musculus* studies can be either *Clinical, Microbial and in vitro* or *Pre-clinical*. However, there are also *M. musculus* studies that are not covered by the existing reporting standards. In this analysis these studies are classified as *Other*. Across the repositories, at the time of analysis, there were no examples of *M. musculus* clinical studies.

As of 17/03/07, there are 33 *M. musculus* studies in MetaboLights and 120 in Metabolomics Workbench. Only studies categorized as pre-clinical were included in this work, as compliance with the *Microbial and in vitro* guidelines was assessed using *H. sapiens* cell line studies. There are 29 *M. musculus* pre-clinical studies in MetaboLights and 91 in Metabolomics Workbench.

### *Arabidopsis thaliana*

The Plant reporting standards are broad and are applicable to all *A. thaliana* studies that were publicly available in the four repositories as of 17/03/07. There were 2 *A. thaliana* studies from MeRy-B, 20 from MetaboLights, 3 from Metabolomics Workbench and 37 from MetaPhen.

### Statistical analysis

For analysing differences within repositories (ML and MW) Kruskal Wallis tests, followed by Dunn *post-hoc* tests with Benjamini-Hochberg correction were used. A Kruskal Wallis test was also used for comparing differences between repositories, both between ML and MW and between all four repositories for the plant guidelines. Mann-Whitney U tests were used to analyse differences between minimal and best practice reporting standards.

### Code availability

All of the analysis was performed using R version 3.3.2. The code used for analysis is available at https://github.com/RASpicer/Compliance_MSI_Guidelines.

## Additional information

**How to cite this article**: Spicer, R.A. *et al.* Compliance with minimum information guidelines in public metabolomics repositories. *Sci. Data* 4:170137 doi: 10.1038/sdata.2017.137 (2017).

**Publisher ’s note**: Springer Nature remains neutral with regard to jurisdictional claims in published maps and institutional affiliations.

## Supplementary Material

Supplementary Information

## Figures and Tables

**Table 1:** Comparison of metabolomics data repositories as of 07/03/17.

Database	Scope	Analysis	No. Studies	No. Public Studies	No. Species
MetaboLights	All Metabolomics	—	435	236	77
Metabolomics Workbench	All Metabolomics	✓	505	365	40
MetaPhen	Plants—GC-MS focused	✓	115	58	17
MeRy-B	Plants—^1^H NMR	✓	54	30	17
The number of species refers to the number of different species across publicly available studies only, and does not include species in private studies.					

**Table 2:** The number of minimal and best practice reporting standards for each biological experimental type.

Standard	Minimal	Best Practice
Mammalian Clinical trials and human studies	22	33
Environmental	22	38
Microbial and *in vitro*	15	39
Plant	20	—
Pre-clinical	30	16
There are no additional best practice reporting standards for plant studies.		
