# Supplementary material for: Compliance with minimum information guidelines in public metabolomics repositories
Source: Sci Data. 2017 Sep 26;4:170137. doi: 10.1038/sdata.2017.137 (PMC5613734; doi:10.1038/sdata.2017.137)
Supplement: Supplementary Information [file sdata2017137-s1.pdf]

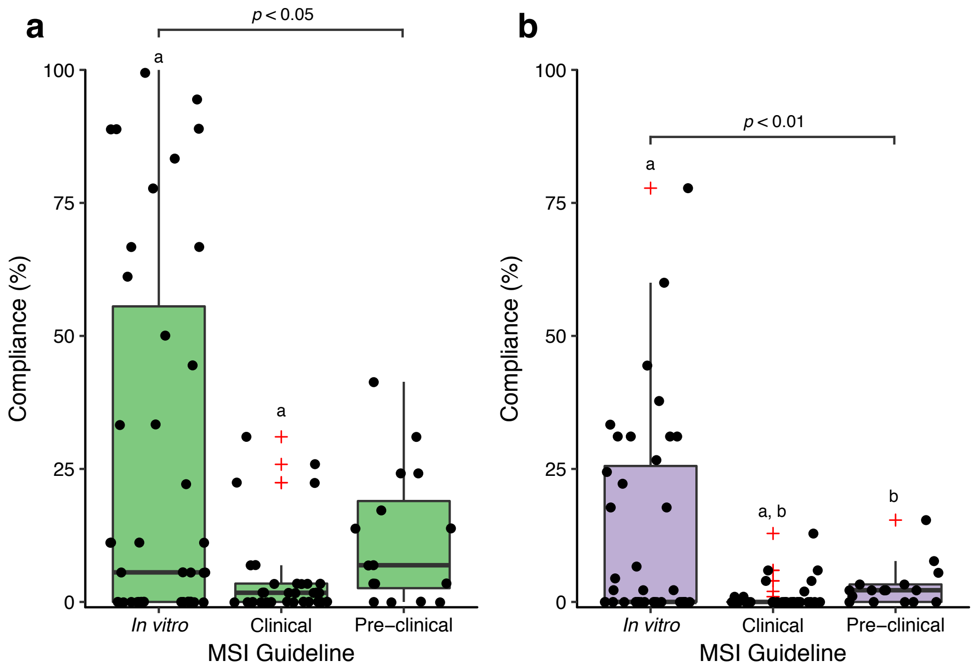

Supplementary Figure 1: Combined box-and-whisker and dot plot showing the percentage compliance with the MSI best practice/optional reporting standards within the a) MetaboLights and b) Metabolomics Workbench repositories. Red '+' indicate outliers. Letters denote significant differences in compliance (Kruskal Wallis, Dunn post-hoc test with Benjamini-Hochberg correction).

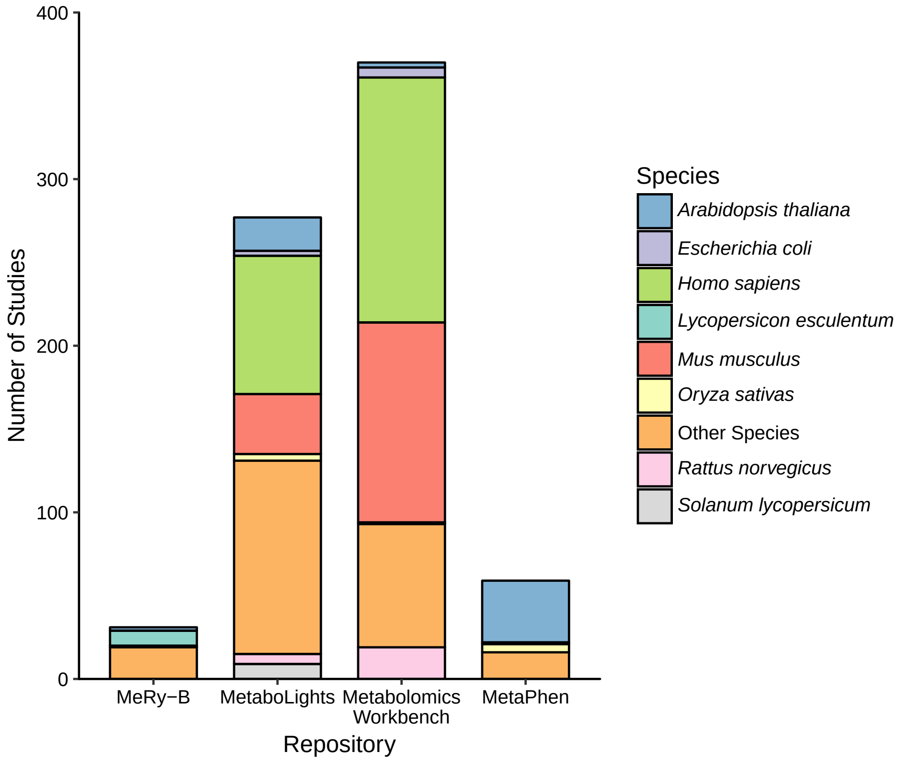

Supplementary Figure 2: The frequency of studies including different species in the metabolomics data repositories: MeRy-B, MetaboLights, Metabolomics Workbench and MetaPhen. For a species to be plotted as an individual band it must have been found in a minimum of nine studies across the repositories. Species found in less than nine studies across the four repositories are reported as Other Species.

Supplementary Table 1: The percentage of *Homo sapiens* studies in each repository that comply with each mammalian clinical trials and human studies minimal reporting standard.

**Minimal Reporting Standard**	**Compliance (%)**	
	**MetaboLights ($n = 58$)**	**Metabolomics Workbench ($n = 99$)**
Biofluid or Tissue	98.28	90.91
Number of Groups	86.21	93.94
Disease Status	60.34	61.62
Sample Storage Temperature	58.62	53.54
Ethical Approval	87.93	15.15
Age Range	81.03	19.19
Weight range and Height and/or BMI	46.55	32.32
Fasting Status	31.03	21.21
Gender	46.55	3.03
Inclusion Criteria	41.38	5.05
Exclusion Criteria	37.93	7.07
Volume or Quantity of Collection	27.59	5.05
Anticoagulant	10.34	10.10
Treatment	3.45	11.11
Ethnicity	6.90	6.06
Trial Type	10.34	1.01
Treatment Dose	3.45	6.06
Location of Collection	6.90	2.02
Treatment Duration	1.72	5.05
Treatment Route	1.72	2.02
Bacteriostatic Agent	0.00	0.00
Treatment Vehicle	0.00	0.00

Supplementary Table 2: The percentage of *Homo sapiens* studies in each repository that comply with each microbial and *in vitro* minimal reporting standard.

**Minimal Reporting Standard**	**Compliance (%)**	
	**MetaboLights (n = 18)**	**Metabolomics Workbench (n = 45)**
Metabolism Quenching Method	83.33	62.22
Harvesting Method	83.33	48.89
Metabolite Extraction	77.78	51.11
Sample Storage	72.22	37.78
Normalisation by Cell Number	5.56	2.22
Cell Integrity	5.56	0.00
Stability	5.56	0.00
Temperature from Sampling to Quenching	0.00	4.44
Extracellular Metabolites Discriminated	0.00	2.22
Recovering from Extraction	0.00	2.22
Time Until Quenching	0.00	2.22
Detection Limit	0.00	0.00
Quality Control	0.00	0.00
Sample Clean-up	0.00	0.00
Sample Storage Duration	0.00	0.00

Supplementary Table 3: The percentage of *Mus musculus* studies in each repository that comply with each pre-clinical minimal reporting standard.

**Minimal Reporting Standard**	**Compliance (%)**	
	**MetaboLights (n = 29)**	**Metabolomics Workbench (n = 91)**
Biofluid or Tissue	100.00	89.01
Number of Groups	75.86	91.21
Strain	100.00	61.54
Treatment	75.86	78.02
Sex	93.10	46.15
Collection Time	89.66	40.66
Age at Collection or Euthanization	68.97	46.15
Sample Storage Temperature	65.52	35.16
Age at Study Start	75.86	24.18
Treatment Duration	72.41	27.47
Animal Supplier	82.76	10.99
Treatment Dose	72.41	19.78
Diet	44.83	40.66
Treatment Route	68.97	14.29
ad lib or Restricted Diet	68.97	6.59
Light Cycle	55.17	9.89
Treatment Vehicle	55.17	6.59
Euthanasia Method	41.38	18.68
Tissue Processing	27.59	25.27
Group or Individual Housing	31.03	5.49
Fasting Status	27.59	8.79
Weight Range	34.48	0.00
Volume or Quantity of Sample Collection	6.90	12.09
Anticoagulant	6.90	0.00
Tap or Purified Water	6.90	0.00
Collection Frequency	3.45	1.10
Collection Method	3.45	1.10
Location of Sample Collection	0.00	2.20
Bacteriostatic Agent	0.00	0.00
Collection Duration	0.00	0.00

Supplementary Table 4: The percentage of *Arabidopsis thaliana* studies in each repository that comply with each plant minimal reporting standard.

**Minimal Reporting Standard**	**Compliance (%)**			
	**MeRy-B (n = 2)**	**MetaboLights (n = 20)**	**Metabolomics Workbench (n = 3)**	**MetaPhen (n = 37)**
Organ or Cell Type	100.00	100.00	100.00	94.59
Plant Growth Stage	100.00	65.00	100.00	78.38
Genotype	100.00	70.00	66.67	100.00
Growth Support	50.00	95.00	66.67	97.30
Light	50.00	90.00	66.67	97.30
Metabolism Quenching Method	100.00	30.00	100.00	64.86
Date(s) of Plant Establishment	50.00	80.00	66.67	89.19
Temperature	50.00	70.00	66.67	78.38
Harvest Time, Date	0.00	65.00	100.00	78.38
Nutrients Regime	50.00	40.00	66.67	64.86
Harvest Method	50.00	30.00	100.00	35.14
Biosource Amount	50.00	65.00	66.67	13.51
Sample Storage	50.00	40.00	66.67	32.43
Humidity	0.00	20.00	66.67	32.43
Treatment	0.00	25.00	33.33	59.46
Treatment Time	0.00	25.00	33.33	56.76
Treatment Dose	0.00	20.00	33.33	59.46
Plot Design	0.00	5.00	66.67	5.41
Growth Location	0.00	15.00	33.33	0.00
Watering Regime	0.00	0.00	33.33	2.70

Supplementary Table 5: The percentage of *Homo sapiens* studies in each repository that comply with each mammalian clinical trials and human studies optional reporting standard.

**Optional Reporting Standard**	**Compliance (%)**	
	**MetaboLights (n = 58)**	**Metabolomics Workbench (n = 99)**
Arterial or Venous Blood	31.03	4.04
Speed of Centrifugation	25.86	6.06
Temperature of Centrifugation	22.41	6.06
Time of Centrifugation	22.41	4.04
Smoking Status	6.90	12.12
Time from Collection to Freezing	6.90	2.02
Drug Consumption	3.45	1.01
Alcohol Consumption	3.45	0.00
Hemoglobin	3.45	0.00
Platelets	3.45	0.00
White Blood Count	3.45	0.00
Creatinine	1.72	0.00
Diet	1.72	0.00
Hemocrit	1.72	0.00
Malnutrition	1.72	0.00
Mid Flow or Total Urine	1.72	0.00
Potassium	1.72	0.00
Sample Storage Duration	1.72	0.00
Sodium	1.72	0.00
Metal Exposure	0.00	0.00
Albumin	0.00	0.00
ALP	0.00	0.00
ALT	0.00	0.00
Bilirubin	0.00	0.00
Glucose	0.00	0.00
-GT	0.00	0.00
HDL Cholesterol	0.00	0.00
Hemolysis	0.00	0.00
LDL Cholesterol	0.00	0.00
Total Cholesterol	0.00	0.00
Total Protein	0.00	0.00
Triglycerides	0.00	0.00
Urea	0.00	0.00

Supplementary Table 6: The percentage of *Homo sapiens* studies in each repository that comply with each microbial and *in vitro* best practice reporting standard.

**Best Practice Reporting Standard**	**Compliance (%)**	
	**MetaboLights (n = 18)**	**Metabolomics Workbench (n = 45)**
Cell Type	100	77.78
Treatment	88.89	60.00
Treatment Dose	88.89	44.44
Medium or Substrate	94.44	37.78
Medium or Substrate Concentration	88.89	31.11
Treatment Time	77.78	33.33
Treatment Vehicle	83.33	26.67
Growth Container	61.11	24.44
Cell Supplier	66.67	17.78
Medium or Substrate Supplier	66.67	17.78
CO_2	50.00	31.11
Temperature	44.44	22.22
Harvesting Time	22.22	31.11
Isotopic Labelling	11.11	31.11
Replicates	33.33	6.67
Subculturing and Splitting Protocols	33.33	0.00
Inoculation Size	11.11	2.22
Growth Support	11.11	0.00
Immortalized or Transformed	11.11	0.00
pO_2	5.56	4.44
pH	5.56	2.22
Additional -omics Datasets	5.56	0.00
Growth Container Supplier	5.56	0.00
Growth Support Supplier	5.56	0.00
Humidity	0.00	2.22
Evaporation	0.00	0.00
Gas Composition	0.00	0.00
Growth Configuration	0.00	0.00
Growth Rate	0.00	0.00
Harvesting Cell Density	0.00	0.00
Harvesting Depletion of Nutrients	0.00	0.00
Harvesting Growth Phase	0.00	0.00
Marker of Differentiated Stage	0.00	0.00
Number of Generations Until Harvesting	0.00	0.00
Number of Culture Passages	0.00	0.00
Pretreatment	0.00	0.00
Pretreatment Time	0.00	0.00
Stabilization Time	0.00	0.00
Stirrer Speed	0.00	0.00

Supplementary Table 7: The percentage of *Mus musculus* studies in each repository that comply with each pre-clinical optional reporting standard.

**Optional Reporting Standard**	**Compliance (%)**	
	**MetaboLights (n = 29)**	**Metabolomics Workbench (n = 91)**
Use of Anesthesia	41.38	7.69
Environmental Enrichment: Temperature	31.03	2.20
Acclimation Duration to Experimental Facility	24.14	3.30
Germ-free or Conventional Housing	24.14	2.20
Fasting Duration	17.24	5.49
Bedding Type	6.90	15.38
Environmental Enrichment: Humidity	13.79	2.20
Anesthesia Time	13.79	0.00
Anesthesia Dose	6.90	2.20
Cage Cleaning Frequency	3.45	0.00
Cage Type	3.45	0.00
Inclusion Criteria	3.45	0.00
Additional Phenotypic Model	0.00	3.30
Sample Storage Duration	0.00	2.20
Temperature of Collection Tube	0.00	1.10
Body Weights or Food Consumption	0.00	0.00
